# Patient reported outcomes in oncology: changing perspectives—a systematic review

**DOI:** 10.1186/s12955-022-01987-x

**Published:** 2022-05-21

**Authors:** Augusta Silveira, Teresa Sequeira, Joaquim Gonçalves, Pedro Lopes Ferreira

**Affiliations:** 1 Health Sciences Faculty, Fernando Pessoa University (UFP-FCS), Rua Carlos da Maia, 296, 4200-150 Porto, Portugal; 2 Centre for Health Studies and Research of University of Coimbra, Centre for Innovative Biomedicine and Biotechnology, Avenida Dias da Silva, 165, 3004-512 Coimbra, Portugal; 3 2Ai - Applied Artificial Intelligence Laboratory, School of Technology of Polytechnic Institute of Cávado and Ave, R. de São Martinho, 4750-810 Vila Frescainha, Barcelos, Portugal; 4 Faculty of Economics, University of Coimbra, Av. Dr. Dias da Silva, 165, 3004-512 Coimbra, Portugal

**Keywords:** Patient reported outcomes, Oncology, Critical success factors, Implementation, Caregivers

## Abstract

In public health context, oncology is associated with severe negative impact on patients and on their relatives’ quality of life. Over the last decades, survival has remained at 50% worldwide for some tumor locations. Patient reported outcomes (PROs) assessment and, the corresponding use in clinical practice, help establishing patient individualized profiling involving caregivers. The purpose of this systematic review was to examine critical success factors for PROs assessment in daily clinical oncology practice. Additionally, we investigated how PROs collection can change oncology perspectives for patients and caregivers. According to PRISMA guidelines, 83 studies were included in this systematic review, whether related with implementation in daily clinical practice or associated with its use in oncology. PROs assessment gathers multi-professional teams, biomedical and clinical expertise, patients, families and caregivers. Institutional involvement, first line for caregiver’s adherence, team continuous formation, encompassing training and support, design of clear workflows, continuous monitoring, and data analysis are crucial for implementation. PROs measures are decisive in oncology. Several items were improved, including caregiver–patient–physician communication, patient risk groups identification, unmet problems and needs detection, disease course and treatment tracking, prognostic markers, cost-effectiveness measurement and comfort/support provision for both patients and caregivers. Routine assessment and implementation of PROs in clinical practice are a major challenge and a paradigm transformation for future.

**Supplementary Information:**

The online version contains supplementary material available at 10.1186/s12955-022-01987-x.

## Introduction

### Global burden of cancer in future

Globally, cancer is the second leading cause of death. In 2018, 18.1 million people worldwide had cancer, and 9.6 million died from this disease. It is expected that cancer cases per year will increase globally, and by 2040, it is anticipated that this figure will nearly double [1]. Moreover, severe negative impact on patients and their relatives’ quality of life (QoL) is also expected. On the other hand, over the last decades, survival has reached 50% worldwide for some tumor locations [2], which naturally increases the impact on patients and families’ experiencing this illness.

Evidence-based interventions focus on early diagnosis and on treatment of curable cancers, as well as on provision of palliative care for all, in order to reduce premature mortality and to optimize QoL. Cancer burden is significant and increasing, involves several domains on individuals, namely physical, social, emotional and cognitive domains, and economical constraints, and it is associated with innumerous negative impacts on communities and on health systems. In addition, a stronger health system should promote stronger cancer care management [1]. However, cancer management is complex and, mainly in this field, the patient should be the keystone. Also, precision medicine ensures better care, enables earlier diagnosis and optimal treatments implemented by multidisciplinary teams, which is expected to guarantee high-quality value-based care. Early detection, quality treatments and patient centered interventions improve both survival rates and QoL, increasing patient confidence and compliance on oncology care [3, 4].

### Patient reported outcomes for patients and caregivers

Patient reported outcomes (PROs) discussion and integration in research and clinical field is not new. PROs measures are multidimensional and subjective, grounded on patient’s perceptions and are objectively quantified. Their use in clinical practice also helps establishing patient individualized profiling involving caregivers. On the other hand, stepped care model based on PROs data collection can be used to predict health outcomes in the future and to take clinical and economic decisions in order to better personalize medical services [2, 4–6].

Recognizing the inclusion of PROs measures in health systems, studies have been conducted to target their implementation in oncology. These measures, including health-related quality of life (HRQoL), capture de voice, thoughts, feelings, and experience during the oncology journey, clearly supporting patients and caregivers, demystifying concepts, clarifying procedures, favoring emotional support, increasing hope, improving communication, helping to create a safer practice environment for patients and providers, and improving patient safety.

They should also integrate advance care planning in life-threatening or life-limiting illness [7–12]. New techniques for remote advance care planning can also take advantage of mobile applications, namely to follow patient wishes by eliciting their individual values and preferences in order to better the quality of the care provided and to reduce their psychological distress [13, 14]. In addition, a recent systematic review showed that administering electronic PROMs may be a good way to perform a good advance care planning [15].

Looking at several reviews, we also evidence the utility of using PRO measures on family caregiver’s support. They are crucial for the optimization of the well-being and HRQoL in oncology and to facilitate patients’ experience. Signalizing specific affected domains that can guide strategies, PROs assessments allow to detect unmet problems and help locate investments with precision [16–19].

However, in research and clinical practice, we should always have evidence that the PROMs administered are reliable and properly validated [20]. We keep in mind that one of the purposes of PROs is to help the decision-making process and so, the measurement instruments used should be appropriate, safe, valid, sensitive and with good psychometric characteristics [21, 22].

The objective of this systematic review was to examine potential critical success factors involved in PROs assessments in oncology clinical practice. Additionally, we investigated how collected PROs scores can modify oncology perspectives for patients and caregivers. All studies were included, whether they report implementations in routine clinical practice or were associated with their use in patient clinical decisions and caregivers’ support. This review also addressed the factors that may affect PROs.

## Materials and methods

The present systematic review examined PROs assessment in oncology clinical practice perspectives and critical success factors for implementation, in accordance with PRISMA guidelines [23, 24]. In this context, implementation is the carrying out, execution, or practice of a plan, a method, for doing something. When considering PROs in oncology, a conceptual definition of implementation means to integrate collection of PROs in routine practice in order to improve personalized oncologic care and support advance care planning.

### Scope of review

This review implemented a systematic methodology to identify critical success factors for PROs measures implemented in routine practice. Its research context encompassed the collection of PROs measures in oncology, the review of PROs intervention and prognostic factors, as well as research articles seen as relevant to health and social care in oncology. Implementation methodology and its influence on the measurement success was also considered.

We did not apply any restrictions related to socio-demographic or clinical characteristics, such as gender, age, ethnicity, tumor location, or TNM classification. However, we excluded other contexts as autoimmune or endocrine diseases, surgery, mental disorders, or chronic pain.

The following question was raised: use PROs in oncology routine clinical practice, does it make any difference? Several domains searched the basis of this question: caregiver–patient–physician communication, patient risk groups identification, unmet problems and needs detection, disease course and treatment tracking, prognostic markers, cost-effectiveness measurement and comfort/support provision for both patients and caregivers.

Also, we searched how PROs assessment gathers multi-professional teams, biomedical and clinical expertise, patients, families, and caregivers. In addition, we searched how institutional involvement, first line caregiver’s adherence, team continuous formation (training and support), design of clear workflows, continuous monitoring, and data analysis does influence routine practice assessment implementation.

### Search strategy

The search was performed on the following electronic bibliographic databases: Pubmed/Medline, PsycINFO, The Cochrane Library, Science Direct and Web of Science (Science and Social Science Citation Index). Grey literature and conference proceedings was also assessed [25, 26], as well as internet resources WHOQOL [27], Multinational Association of Supportive Care in Cancer (MASCC) [28], and International Society for Quality of Life (ISOQOL) [29, 30].

The search strategy allowed us to find published and unpublished studies. We only considered terms relating to or describing the intervention (PROs implemented and used in clinical practice). It included terms as oncology patients, caregivers, clinical decision-making, stepped care model, predictive prognosis, patient centered care, critical successful factors related to implementation of PRO measures, daily clinical practice, routine assessment, and electronic tools. These terms were combined with the Cochrane Medline filter for controlled trials of interventions.

There were no language restrictions and we focused on studies published between January 2011 and 2021 (10 years).

Initially, any type of article was considered eligible, including systematic reviews, research articles, or prospective and retrospective studies. This means that it was included any paper (1) reflecting the critical success factors considered for the intervention, (2) using electronic tools to optimize the implementation of systematic assessment of PROs in oncology clinical practice; (3) analyzing change perspectives in oncology clinical practice related to the use of PROs; or (4) addressing health and social care in oncology.

However, some studies were excluded, namely if (1) reporting implementation in other health chronic conditions rather than oncology; (2) were duplicated; (3) with full text not available, or (4) were not the original article.

### Study quality assessment, data extraction, and analysis plan

All articles searched were filtered using broad selection criteria framed as questions:

- “Does the article address any aspect of PROs implementation and use in oncology?
- “Is the article relevant to PROs collection and cancer?”

The study selection and data extraction were blinded and, after the search, all references were sent to a reference management system (Mendeley). Duplicate articles were removed, and the titles and abstracts of the remaining articles were evaluated.

To identify eligible studies, three other questions were raised and answered: (1) was the topic related with the defined scope? (2) did it fit the inclusion and exclusion criteria? and (3) was the methodology appropriate?

A standardized pre-piloted form was used to extract data from the included studies in order to obtain an extraction process as comprehensive, transparent, and objective as possible. Two reviewers extracted data independently, and discrepancies were identified and resolved through discussion with a third author. This third reviewer scrutinized 10% of the data to verify the consistency of the extraction process and to solved discrepancies. Other CEISUC researchers were also contacted to provide missing or additional data [25, 26].

Each article was graded as having low, moderate or high relevance. Articles were considered of high interest whenever they effectively demonstrated impact on the considered items, and moderate those that prospected such items. Low relevance was attributed to all manuscripts that did not present any conclusions or perspectives in these domains.

Data from scoping literature were extracted into an electronic Excel data sheet and constructed by using a support checklist. The datasheet was divided into sections, with each section dedicated to a theory, area, concept, theme, or element from the framework of PROs intervention. After synthesizing the data and assessing the quality of the evidence, the writing of the systematic review article begun.

## Results

### Process selection

**Fig. 1:**
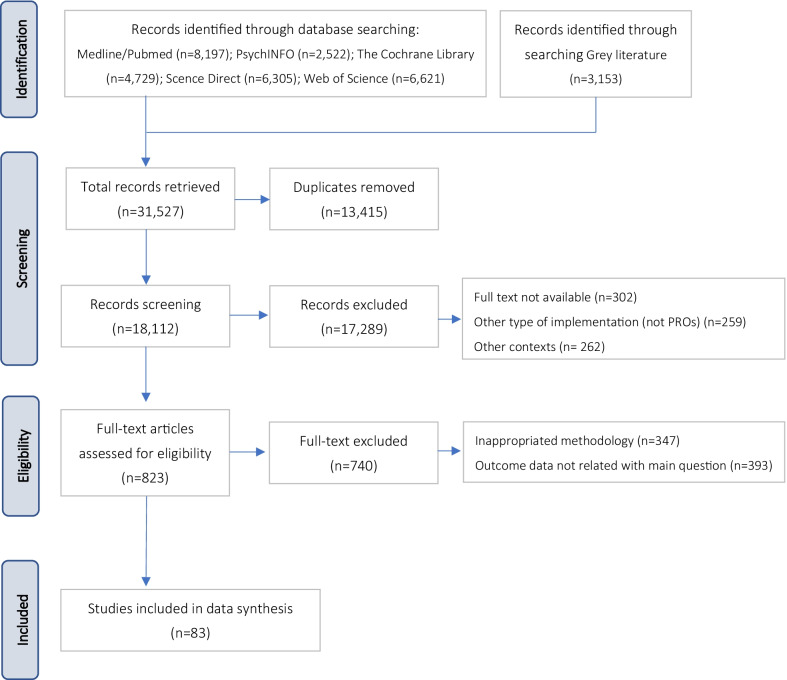
PRISMA flow diagram

Figure 1 presents the PRISMA flowchart detailing record identification, selection, eligibility, and inclusion.

A total of 31,527 records were retrieved from the five data bases and grey literature. After removing the duplicated and the records not fulfilling the inclusion criteria, we came up with 83 records.

### Study characteristics

Summary characteristics of these 83 selected articles are synthetized in Table 1 and, in Additional file 1: Table S1, we present the main characteristics of the articles.

**Table 1:** Study characteristics of all studies (N = 83)

Characteristics	Classification	N (%)
Study design	Systematic review (SR)	27 (32.5%)
	Research article (RA)	21 (25.3%)
	Retrospective, prospective (RP)	35 (42.2%)
Based on HRQoL		47 (56.6%)
Oncology perspectives interest	High (H)	70 (86.4%)
	Moderate (M)	11 (13.6%)
	Low (L)	0 (0.0%)
Oncology implementation interest	High (H)	55 (88.7%)
	Moderate (M)	7 (11.3%)
	Low (L)	0 (0.0%)

Based on study design, most studies were retrospective or prospective (n = 35, 42.2%) and a minority of them were research articles (n = 21, 25.3%). All studies reported PROs and most studies specifically explored HRQoL (n = 47; 56.6%). Studies were also classified into two main domains: PROs applied to oncology perspectives (n = 81; 97.6%) and PROs applied to oncology implementation (n = 62; 74.7%). Overall, the vast majority of studies were considered of high interest according to the methodological criteria—either for oncology perspectives or for oncology implementation (86.4% and 88.7%, respectively). Articles graded as with low relevance were excluded.

Table 2 summarizes the characteristics of the 83 articles selected.

**Table 2:** Selected articles type and rated interest considering the published year (%)

Type of article	2013	2014	2015	2016	2017	2018	2019	2020	2021
HQoL	14.3	14.3	37.6	20.0	29.4	22.5	17.9	20.5	30.8
Systematic reviews	14.3	21.4	0.0	30.0	14.7	10.0	12.8	5.1	15.4
Research	0.0	0.0	18.8	0.0	11.8	7.5	12.8	7.7	7.7
Retrospective and prospective	19.1	21.4	25.0	10.0	8.8	20.0	15.4	31.0	7.7
High interest	19.1	35.7	31.3	30.0	17.6	32.5	23.1	33.3	38.5
Moderate interest	9.5	7.1	8.3	10.0	17.6	5.0	17.9	5.1	0.0

HRQoL and retrospective and prospective articles were found to be associated to higher scores; high interest rated articles were more frequent along the years. We also observed an increase in articles related to HRQoL and a decrease in articles considered as research articles. However, despite the sample differences verified, a qui-square test did not confirm any significance of the differences observed.

### PROs oncology perspectives

*A* total of 81 (97.6%) studies investigated whether PROs collection determined advantages for patients (n = 67; 87.7%), caregivers (n = 21; 25.3%) and health systems, communities and society (n = 63, 75.9%), as shown in Table 3.

**Table 3:** PROs: oncology perspectives

	N (%)	Perspectives	N (%)
Oncology patients	67 (87.7%)	Stepped care models	28 (41.8%)
		Prognosis value	34 (50.7%)
		Communication	37 (55.2%)
		Patient safety	40 (59.7%)
		Risk identification	50 (74.6%)
Caregivers	21 (25.3%)	Communication improvements	7 (33.3%)
		Supportive care guidance	7 (33.3%)
		Caregiver support and unmet needs identification	14 (66.7%)
		Caregiver burden	16 (76.2%)
Health systems, communities and society	63 (75.9%)	Futures perspectives in oncology	40 (63.5%)
		Economical decisions, cost-effective measures	4 (6.3%)
		Drug approval	4 (6.3%)
		Patient centered care	51 (81.0%)

The percentages’ sum can be higher than 100% since some articles addressed several domains

When studying PROs oncology patient perspectives, the most expressive association occurred between PROs collection and risk identification (n = 50; 74.6%). In addition, the contribution to stepped care models design revealed the lowest association (n = 28; 41.8%). When considering PROs caregivers’ perspectives, studies investigated four main positive effects: (1) caregiver burden measurement (n = 16; 76.2%), (2) family support (n = 14; 66.7%), (3) supportive guidance (n = 7; 33.3%), and (4) communication improvements (n = 7; 33.3%). Studies reporting PROs interest for healthcare systems, communities and society, highlighted their contribution to patient-centered care (n = 51; 81.0%), how PROs can change future perspectives in oncology (n = 40; 63.5%) and, in a lesser extent, their collaboration on drug approvals (n = 4; 6.3%), economical decisions and cost-effective measures (n = 4; 6.3%).

### PROs oncology implementation

A total of 62 studies investigated PROs implementation in routine clinical practice (74.7%). A clear identification of critical success factors was present in 23 studies (27.7%) and the methodology description of implementation was described in 22 (26.5%), as identified in Table 4.

**Table 4:** PROs implementation perspectives

	N (%)	Perspectives	N (%)
Critical success factors	23 (27.7%)	Staff involvement	17 (73.9%)
		Institution approval	11 (47.8%)
		Clear workflows	18 (78.3%)
		Team continuous formation	12 (52.2%)
		Data analysis	16 (69.6%)
Implementation methodology	22 (26.5%)	Research intervention	12 (54.5%)
		Clinical interventions	20 (90.9%)
		Medical interventions	13 (59.1%)
		Physiological interventions	8 (36.4%)
		Social interventions	2 (9.1%)
Routine clinical practice	62 (74.7%)	Use for clinical purposes	46 (74.2%)
		Use for research purposes	19 (30.6%)
		Use for economical purposes	8 (12.9%)
		Quality of care	49 (79.0%)

The percentages’ sum can be higher than 100% since some articles addressed several domains

The most frequently identified critical factors for successful implementation were the clear workflows definition (n = 18; 78.3%) and staff involvement importance (n = 17; 73.9%), being the one with the lowest expression the institution approval (n = 11; 47.8%). On the other hand, implementation methodology was most often considered for clinical interventions (n = 20; 90.9%) and less considered for social purposes (n = 2; 9.1%). At last, routine clinical practice mostly described interventions for quality of care (n = 49; 79.0%) and clinical purposes (n = 46; 74.2%), while economical purposes (n = 8; 12.9%) was the least encountered. Table 5 reveals the amount (%) of articles found for each perspective considered in Tables 3 and 4.

**Table 5:** Proportion of articles found for each perspective considered and assigned interest (%)

	HRQoL		Systematic reviews		Research		Retrospective and protective		High interest		Moderate interest	
	Yes	No	Yes	No	Yes	No	Yes	No	Yes	No	Yes	No
*Oncology perspectives*												
Oncology patients												
Stepped care models	32.4	37.7	27.6	48.0	42.2	5.3	32.6	35.0	18.2	39.3	38.7	19.0
Prognosis value	26.5	51.0	41.4	40.0	45.3	26.3	34.9	47.5	27.3	49.5	45.5	28.6
Communication	44.1	44.9	46.6	40.0	51.6	21.1	34.9	55.0	22.7	52.5	51.6	28.8
Patient safety	52.9	44.9	53.4	36.0	51.6	36.8	39.5	57.5	27.3	55.7	54.8	28.6
Risk identification	50.0	67.3	63.8	52.0	65.6	42.1	51.2	70.0	50.0	63.9	62.9	52.4
Caregivers												
Communication improvements	8.8	8.2	10.3	4.0	6.3	15.8	7.0	10.0	9.1	8.2	8.1	9.5
Supportive care guidance	11.8	6.1	10.3	4.0	7.8	10.5	4.7	12.5	9.1	8.2	8.1	9.5
Caregiver support and unmet needs identification	17.6	16.3	24.1	0.0	7.8	47.7	16.3	17.5	27.3	13.1	14.5	23.8
Caregiver burden	17.6	20.4	24.1	8.0	14.1	36.8	18.6	20.0	27.3	16.4	16.1	28.6
Health systems												
Futures perspectives in oncology	50.0	46.9	51.7	40.0	43.8	63.2	46.5	50.0	27.3	55.7	56.5	23.8
Economical decisions, cost-effective measures	8.8	2.0	3.4	8.0	6.3	0.0	4.7	5.0	4.5	4.9	4.8	4.8
Drug approval	5.9	4.1	1.7	12.0	6.3	0.0	7.0	2.5	0.0	6.6	6.5	0.0
Patient centered care	64.7	59.2	63.8	56.0	65.6	47.4	51.2	72.5	50.0	65.6	64.5	52.4
*Implementation perspectives*												
Oncology patients												
Staff involvement	20.6	20.4	24.1	12.0	21.9	15.8	14.0	27.5	0.0	27.9	27.4	0.0
Institution approval	14.7	12.2	19.0	0.0	15.6	5.3	2.3	25.0	0.0	18.0	17.7	0.0
Clear workflows	20.6	22.4	31.0	0.0	23.4	15.8	9.3	35.0	0.0	29.5	29.0	0.0
Team continuous formation	14.7	14.3	20.7	0.0	15.6	10.5	4.7	25.0	0.0	19.7	19.4	0.0
Data analysis	17.6	20.4	25.9	4.0	21.9	10.5	7.0	32.5	0.0	26.2	25.8	0.0
Oncology patients												
Research intervention	8.8	18.4	20.7	0.0	15.6	10.5	4.7	25.0	0.0	19.7	19.4	0.0
Clinical interventions	29.4	20.4	29.3	12.0	26.6	16.8	14.0	35.0	9.1	29.5	29.0	9.5
Medical interventions	14.7	16.3	22.4	0.0	17.2	10.5	4.7	27.5	0.0	21.3	21.0	0.0
Physiological interventions	5.9	12.2	13.8	0.0	10.9	5.3	2.3	17.5	4.5	11.5	11.3	4.8
Social interventions	0.0	4.1	3.4	0.0	1.6	5.3	2.3	2.5	0.0	3.3	3.2	0.0
Oncology patients												
Use for clinical purposes	55.9	55.1	56.9	52.0	57.8	47.4	48.8	62.5	27.3	65.6	66.1	23.8
Use for research purposes	20.6	24.5	27.6	12.0	23.4	21.1	16.3	30.0	4.5	29.5	29.0	4.8
Use for economical purposes	8.8	10.2	6.9	16.0	10.9	5.3	11.6	7.5	0.0	13.1	12.9	0.0
Quality of care	58.8	57.1	70.7	28.0	54.7	68.4	41.9	75.0	59.1	57.4	58.1	57.1

To find out if the differences found in the sample were significant, a qui-square test for comparison of proportions was performed. It was possible to confirm that the differences observed in research articles regarding stepped care models, communication, caregiver support & unmet needs identification, and caregiver burden were significant, i.e., these articles were less related to cancer patients and their caregivers.

Regarding retrospective and prospective articles, the same test revealed that there was a lower incidence of this type of articles when considering institution approval, clear workflows, team continuous formation, data analysis, research intervention, clinical interventions, medical interventions, or physiological interventions and quality of care. Thus, articles that refer to oncology implementation, especially regarding “critical success factors” and “implementation methodology”, appeared to a lesser extent in retrospective and prospective articles.

Moreover, manuscripts considered to have a higher level of interest revealed a higher proportion in articles related to cancer patients.

## Discussion

### PRO in oncology: changing perspectives

PROs may become a new center of influence in oncology, affecting both oncology quality of care and patient satisfaction. Their routine integration in oncology clinical practice and patients’ involvement in the disease course affected, in a decisive manner, the process of oncology care, understanding benefits and risks of a proposed treatment, and weighing the impact of a decision on symptoms, function, and life expectancy [31]. PROs collection and its incorporation in routine clinical practice must improve patient’s compliance, which can exceed 80% in daily routine care [31–35].

### PROs: oncology patients

Five main advantages related with cancer patients are consistently and actually presented in literature regarding PRO incorporation in daily clinical practice. They are: (1) stepped care models design reflecting patient’s disease pathway [4, 5, 31, 32, 34, 36–57] (2) prognosis value [2–4, 22, 31, 34, 37–43, 47, 50, 53, 57–74] (3) communication improvements [4, 5, 10, 22, 31, 32, 34, 36, 38, 40–43, 46–50, 53, 55, 65–69, 71–82], (4) patient safety optimization [4, 5, 10, 30, 31, 34, 36, 39–43, 47, 48, 52, 53, 55, 56, 60–62, 64–66, 68, 70, 72–74, 77–87] and (5) healthcare risk identification, i.e., unmet problems identification, symptom detection and symptom control [2, 4, 5, 10, 12, 22, 31, 32, 34, 36, 37, 39–43, 45, 46, 49–51, 53, 55, 56, 58–62, 64–66, 68, 69, 71–82, 84, 88–90].

The present review identified parameters that appear to be most consistently associated with real changes in oncology. Risk identification (74.6%) and patient safety (59.7%) were the most evident topics, followed by communication improvements (55.2%) and prognosis value of PROs (50.7%) [32, 34]. Prior reviews highlighted that adverse events and medical errors are substantially related to communication breakdown and without mentioning PROs, and that clinical manifestations are considerably underestimated, hampering high-risk patients’ identification and patient safety [91]

Indeed, most studies reviewed the evidence that the communication processes in health systems are complex. PROs incorporation in oncology improves clinical practice [42, 47, 92], structures the communication, develops effective communication tools and communication skills training [93–95], and contributes to the delivery of effective oncology high-quality care [34, 39, 76], improved patient-safety and patient satisfaction, and reduces patient anxiety [39, 42, 48, 49, 65–67, 77, 88].

The prognostic value of baseline and follow-up PROs, as independent predictor of the overall survival and likelihood of hospitalization, has been highlighted in randomized clinical trials and observational "real-world" cohort studies [37, 59, 61]. Mierzynska et al*.,* in a systematic review updating a previous one, revealed that 93% of the trials consulted exposed at least one PRO domain as independently prognostic [59]. PROs have higher prognostic value than the one provided by clinical and sociodemographic variables. For cancer populations at various disease stages, PROs and traditional predictors increase the overall survival prediction ability by 6%, when compared to the traditional information used alone or together in multivariable analyses. A systematic review protocol published by Deliu et al*.* identified key areas of improvement and confirmed that well-conducted and reported prognostic model studies with a PRO predictor have great potential to improve healthcare delivery [38]. The physical functioning, global health and QoL domains were consider significant prognostic factors (prognosticators) and several domains have been evidenced [39, 40, 43] including patient-reported functional scores [37, 42, 60, 61, 63, 68], patient-reported symptom interference [38, 49, 59, 62, 65, 66], patient-reported depression [96], or patient-reported fatigue [67, 69].

Stepped care models design based on PROs data collection can reflect the patient’ disease pathway, allowing the development of appropriate and sustainable long-term follow-up models for cancer survivors, foreseeing supported self-management and shared care (supportive care, epidemiological data, groups screening) [5, 32, 34, 36, 37, 40, 41, 43, 47, 49, 54, 56, 57, 65, 97]. Automated algorithms may support risk-stratified guideline-informed care [4, 31, 38, 42, 47, 50, 52, 53].

The present study reports that PROs can capture patient’s essentials on disease pathway. They are crucial at baseline and follow-up stages and consistently consider them in research and clinical practice is a major concern for future in health systems [31–34, 37–42, 50, 51, 59–63, 65, 66, 69, 77, 88, 93, 94, 97–99].

### PROs: oncology caregiver and families

A body of literature [70, 83, 84, 98, 100–107] consider four main topics about this subject: (1) communication improvements [16, 43, 89, 100, 108, 109], (2) supportive care guidance [16, 43, 84, 100, 102, 108], (3) caregivers support and families’ unmet needs identification [12, 16, 17, 84, 89, 100, 102–106, 109], and (4) caregiver burden [12, 16, 17, 83, 85, 89, 98, 100, 102, 104, 107, 108]. The latter had the largest expression in our study (76.2%). This result is understandable if we admit that it actually represents the convergence of the different identified contributions [36, 98, 103, 107, 110].

The roles and the responsibilities of informal caregivers (family members or friends), caring for dependent patients, are also a strong help and invaluable support to oncology patients. Caregivers are influencers of quality of cancer on multiple levels. The impact of this engagement on patients’ lives and overall well-being needs to be measured, and literature reveals the need to develop instruments to better capture this issue. The authors also signalize that only few measures have been subject to psychometric evaluation in cancer caregivers. Shilling et al*.* in their systematic review identify instruments that measure the impact of caregiving and identify publications evaluating psychometric performance in the target population. They concluded that it was not possible to consider the performance of the measures across a group of studies as several domains were not well captured and measures needed to be adapted to current days because, some of them, have already 35 years [51, 98, 100].

Assuming that the experience of caring is multigenerational, literature revealed physical, psychosocial and emotional problems with multidimensional impacts on caregiver experience, and pointed out that some domains are poorly captured. These included changes on career aspiration and planning, paid employment and financial burden, sexual activity, as well as in roles and responsibilities, and family as a unit. Silveira et al*.* in 2018 concluded that being 18–30 or 46–60 years old, being a woman and having low education increase caregivers’ risk factors to poor QoL related to care experience. All scores were worse in caregivers who cared for more than six hours a day [16].

The majority of all selected studies revealed that PROs assessment improves caregiver’s comfort, support [16, 89] and global QoL (66.7%). Their perspectives and perceptions, systematically assessed and implemented at the earliest stages of the oncology process, allow to identify multidimensional problems about caregiver burden, reveal early caregiver unmet needs (60–70% of caregivers), and promote their QoL [43, 110]. This also may contribute to optimize strategies to monitor and implement caregivers’ supportive care or to support interventions and health protection, including physical activity, nutritional intervention, behavioral and cognitive-behavioral therapy, psycho-education interventions, caregiver skill training, couples therapy, decision support, mindfulness-based stress reduction and goal management therapy [102, 103, 111].

On the other hand, communication constraints potentially exacerbate caregivers’ distress, but communication in oncology context, remains a challenge. Effective communication for caregivers is essential to reduce caregiver burden, promoting confidence and intimacy and improving caregiver and families’ QoL [16, 98, 100, 102, 108].

Each caregiver develops his/her own type of conversation based on family patterns, self-efficacy, ethnic origin, duration of care and socio-demographic characteristics. Wittenberg et al*.* in 2017 presented four caregiver types based on patterns of family conversation, considering high/low levels of conformity and conversation: manager, provider of care, self and partner. They argue that PROs can provide detailed and personalized information about each type of caregiver communication, optimizing open and person-centered communication in oncology, content (illness, emotions, daily life, death, sexuality), style (language, atmosphere), timing and preferences [105].

### Health systems, communities and society

Oncology care is a dynamic and multidimensional healthcare that evolves complex economical and clinical decision making and requires demanding care coordination [31, 47, 52, 85]. PRO may become a new center of influence in oncology for health systems, communities and society**,** supporting research directions and cancer comparative effectiveness research, political decisions, strategy and delivery of healthcare, funding and commissioning [31, 43, 52, 60, 65, 66, 112]. In fact, patient-centered care was the most reported domain in our study (81.0%), followed by futures perspectives in oncology (63.5%).

A significant financial burden for health care system is associated to cancer. Kerrigan et al*.* refer that 55% of the costs are comprised in patient hospitalization, increasing steeply in the last month before death. PROs incorporation in clinical practice has been shown to reduce healthcare costs, outpatient visits, hospital admissions and the number of emergency events [60].

PROs are now included by the US Food and Drug Administration (FDA) among the four types of clinical outcomes assessment measures that can be used to determine whether drugs provide a treatment benefit. HRQoL is included on the National Cancer Institute Common Terminology Criteria for Adverse Events scale to detect adverse events and define the doses. This reveals a growing interest and recognition by international health policy and regulatory authorities. PRO measures are relevant to acquirement, development and large-scale application of clinical outcome assessments in health care practice, regulatory and population or surveillance settings, program evaluations, case studies and economic analyses [38, 47, 86]. PROs incorporation in clinical practice are helping physicians in the decision-making process, allowing the assess to patient medication and guiding targeted cancer interventions both for patients and caregivers, reducing costs and increasing effectiveness, satisfaction and outcomes [7, 32, 37, 43, 59, 60, 67, 113]. As MASCC advocates, supportive care makes excellent cancer care possible [28].

### PROs in oncology: implementation

PROs reflect patients’ perceptions, such as health status, symptoms, functioning, satisfaction, health behaviors, and QoL. They are multidimensional and subjective measures, standardized and objectively quantified. PROs are also complementary tools to “the black and white measures”, usually tested and recorded by healthcare providers, such as survival, morbidity, recurrence rates, objective tumor response or disease remission [22, 114]. PROs were firstly addressed in retrospective studies but, for researchers and healthcare providers, the most important challenge was PROs incorporation in clinical practice, integrating the clinical protocols and using them on daily clinical decisions to personalize oncologic clinical care. Studies describe worldwide implementation experiences, carried out in the last 20 years. Linendoll et al.’s systematic review revealed in 2016 that half of the studies included where conducted after 2005, demonstrating a growing interest in oncology PROs [58]. Implementation use into routine clinical practice is feasible and practicable [22, 42, 43, 47, 49, 53, 58, 71, 78, 90, 115–118].

### Critical success factors for implementation in routine clinical practice

Implementation strategies do impact implementation success. A number of critical success factors have been identified and should be considered when planning the strategy. Literature reflects that researchers, policymakers, healthcare professionals, patients and caregivers generally accept the dare of using PROs in real time, making available the big dream of including actual patients’ perceptions on decision-making [22, 47, 48, 54, 71, 78, 90, 114–116]. The authors consider that it is essential to identify critical success factors for implementation in daily clinical practice and to deal with barriers. Ahmed et al*.* considered that PROs, as transformers of the health care system, to be more patient-centered, is still aspirational, revealing that future remains defiant [43].

A majority of all selected studies reveals that robust technology support incorporated across the patient care spectrum will be essential in the future to overcome serious technical and logistic problems, allowing the use of PROs scores on the shortest time, ideally just a few minutes after questionnaires completion [50, 55, 71–73, 78–82, 97, 108, 116]. Our results highlight that this is a real need in future perspectives, linking PRO and oncology.

A properly developed computer platform is imperative for questionnaires completion. It allows data collection by self-administration, database construction, development of evidence-based algorithms, including computerized alerts for symptoms and reminders, providing also documentation templates and PROs graphic reports with reference information and clinical utility [9, 50, 97].

PROs depend on rigorous quality of the collection and management of patient-reported data. Appropriate questionnaires must be properly selected. However, some PROs result from adaptations and some categories do not reflect patients concerns, being required more patients’ active involvement and engagement in PRO measures development. The choice of the most adequate measures for the population of interest provides a plan to minimize potential bias, improving sensitive analysis and clinical utility. The development of digital platforms, which are repositories of translated and validated general and specific instruments is essential for its excellent exposure and selection [22, 25, 26, 77, 119].

Stakeholder engagement and institutional support have also been proposed has decisive steps to accelerate PROs implementation, proactively determining PROs as a priority, including their use in daily clinical practice, scaling costs, facilitating logistics and analyzing cost–benefit especially related to quality assessment sustainability and practice transformation [10, 22, 56, 77, 87, 120]. Because healthcare professional’s involvement is crucial, clinical workflows should be considered on PROs assessment implementation, a topic highlighted in our study.

Providers’ concerns must be discussed as well. From the literature, among the major worries are time spending, measure harmonization, user-friendly data displays, PROs use in clinical practice, PROs trust and utility, redundant work, reduced face-to-face interaction, impact on patient-clinician relationships and confidentiality. When professionals are involved into interactive training courses to assess PROs concepts, informatic platforms functioning or results interpretation, they express highest agreement for their relevance in oncology clinical practice, its viability and its usefulness as a health education tool and, above all, as a promoter of patient-centered healthcare [77, 90, 121, 122].

The PROs implementation in oncology can be complex and challenging. Three phases need to be considered: pre-implementation, implementation, and post-implementation. The continuous monitoring of the process, after initial and successful implementation, is really needed. All barriers must be solidly and continually identified, quantified and discussed to be solved. Oncology patients must consider PROs collection as part of the clinical approach and be sure that someone will use these outcomes to design clinical and research approaches, to improve their health and to optimize their QoL. Patient´s voice is crucial to oncology care delivery, especially when low health literacy is considered. Only PROs systematic collection makes that voice audible and a decisive influence in health context [42, 43, 49, 53, 57, 74, 77, 90, 123, 124].

### Limitations and future perspectives

There are some limitations to the presented study. First, the study presents advantages on using PROs in oncology clinical practice and implementation concerns separately, but frequently studies bring together these two aspects. Second, implementation experiences are world widely related to general chronic diseases, not only in oncology context. However, some differences may also appear, mainly related with cancer health providers and oncology clinical workflows. Third, we did not evaluate if studies used any implementation science theories [125]. At last, mathematical models and informatics concerns were moderately considered.

Future approaches should demystify concepts, strengthen multi-professional approaches from researchers, clinicians, informatics, and mathematicians, and build robust, reliable, and adequate measures. They should also include consultations with patients or members of the public, whose importance is highlighted in the literature [126]. On the other hand, implementations models should be more common in health organizations around the world and presented systematically to scientific community.

## Conclusions

PROs are decisive for patients and caregivers support in oncology. Several items were improved, including caregiver-patient-physician communication, patient risk groups identification, unmet problems and needs detection, disease course and treatment tracking, prognostic markers, cost-effectiveness measurement, and comfort or support provision for both patients and caregivers.

Critical success factors were identified, being recognized that PROs assessment gathers multi-professional teams, biomedical and clinical expertise, patients, families and caregivers. On the other hand, institutional involvement, first line caregiver’s adherence, team continuous formation encompassing training and support, design of clear workflows, continuous monitoring, and data analysis are crucial for daily practice assessment implementation.

In addition, stepped care models based on patients’ stratification and perceptions are the keystone to better personalize medical services. Patient centered care doesn’t exist without patients’ perceptions and participation in clinical decisions. PROs measures are also decisive for predictive prognosis and effective economical decisions.

Routine assessment and implementation of PROs in oncology clinical practice are a major challenge and a paradigm transformation for future.

## Supplementary Information

**Additional file 1.** Summary of studies’ characteristics.

## Data Availability

The dataset used and/or analysed during the current study is available from the corresponding author on reasonable request.

## Abbreviations

EQ-5D-5L: Euroqol 5dimensions 5 levels
GImQ: Headache Impact Questionnaire
HADS: Hospital Anxiety and Depression Scale
ICC: Intraclass correlation coefficient
KMO: Kaiser–Meyer–Olkin
MIDAS: Migraine Disability Assessment Scale
mMIDAS: Modified Migraine Disability Assessment Scale
mMIDAS-P: Portuguese mMIDAS
QoL: Quality of life
VAS: Visual Analogue Scale

## References

[1] World Health Organization. WHO report on CANCER: settings priorities, investing wisely and providing care for all. https://www.who.int/publications/i/item/who-report-on-cancer-setting-priorities-investing-wisely-and-providing-care-for-all.

[2] Sequeira T, Sousa G, Monteiro E, Silveira A (2020). Inflammatory pathology of the oral cavity: relevance in oral oncogenesis. Acta Sci Dent Sci.

[3] Shrestha A, Martin C, Burton M, Walters S, Collins K, Wyld L (2019). Quality of life versus length of life considerations in cancer patients: a systematic literature review. Psychooncology.

[4] Silveira A, Monteiro E, Sequeira T (2020). Head and neck cancer, precision medicine and health related quality of life: the patient is the keystone. J Head Neck Spine Surg.

[5] Tran K, Zomer S, Chadder J, Earle C, Fung S, Liu J, Louzado C, Rahal R, Shaw Moxam R, Green E (2018). Measuring patient-reported outcomes to improve cancer care in Canada: an analysis of provincial survey data. Curr Oncol.

[6] Deshpande PR, Rajan S, Sudeepthi BL, Abdul Nazir CP (2011). Patient-reported outcomes: a new era in clinical research. Perspect Clin Res.

[7] Haraldstad K, Wahl A, Andenæs R, Andersen JR, Andersen MH, Beisland E, Borge CR, Engebretsen E, Eisemann M, Halvorsrud L, Hanssen TA, Haugstvedt A, Haugland T, Johansen VA, Larsen MH, Løvereide L, Løyland B, Kvarme LG, Moons P, Norekvål TM, Ribu L, Rohde GE, Urstad KH, Helseth S, LIVSFORSK network (2019). A systematic review of quality of life research in medicine and health sciences. Qual Life Res.

[8] Zwakman M, Jabbarian LJ, van Delden J, van der Heide A, Korfage IJ, Pollock K, Rietjens J, Seymour J, Kars MC (2018). Advance care planning: a systematic review about experiences of patients with a life-threatening or life-limiting illness. Palliat Med.

[9] Broomfield K, Harrop D, Judge S, Jones G, Sage K (2019). Appraising the quality of tools used to record patient-reported outcomes in users of augmentative and alternative communication (AAC): a systematic review. Qual Life Res.

[10] Austin E, LeRouge C, Hartzler AL, Segal C, Lavallee DC (2020). Capturing the patient voice: implementing patient-reported outcomes across the health system. Qual Life Res.

[11] de Bienassis K, Kristensen S, Hewlett E, Roe D, Mainz J, Klazinga N (2021). Measuring patient voice matters: setting the scene for patient-reported indicators. Int J Qual Health.

[12] Ringash J, Bernstein LJ, Devins G, Dunphy C, Giuliani M, Martino R, McEwen S (2018). Head and Neck Cancer Survivorship: Learning the Needs. Meeting the Needs Semin Radiat Oncol.

[13] Bell JG, Shaffer LE, Schott VA, Reynolds K, Elliott J, Secic M, Aldrich ER, Taylor C, McMath T (2020). Patient navigation effect on cancer patients’ quality of life and distress. J Acad Oncol Nurse Patient Navig.

[14] Kamran R, Dal Cin A (2020). Designing a mission statement mobile app for palliative care: an innovation project utilizing design-thinking methodology. BMC Palliat Care.

[15] Huber MT, Highland JD, Krishnamoorthi VR, Tang JW (2018). Utilizing the electronic health record to improve advance care planning: a systematic review. Am J Hosp Palliat Care.

[16] Silveira A, Amaral S, Castro AR, Monteiro E, Pimentel F, Sequeira T (2018). Cancer palliative care: technology support for quality of life assessment of family caregivers. Procedia Comput Sci.

[17] Ullrich A, Ascherfeld L, Marx G, Bokemeyer C, Bergelt C, Oechsle K (2017). Quality of life, psychological burden, needs, and satisfaction during specialized inpatient palliative care in family caregivers of advanced cancer patients. BMC Palliat Care.

[18] Yu H, Li L, Liu C, Huang W, Zhou J, Fu W, Ma Y, Li S, Chang Y, Liu G, Wu Q (2017). Factors associated with the quality of life of family caregivers for leukemia patients in China. Health Qual Life Outcomes.

[19] Krug K, Miksch A, Peters-Klimm F, Engeser P, Szecsenyi J (2016). Correlation between patient quality of life in palliative care and burden of their family caregivers: a prospective observational cohort study. BMC Palliat Care.

[20] Mokkink LB, Terwee CB, Patrick DL, Alonso J, Stratford PW, Knol DL, Bouter LM, de Vet HC (2010). The COSMIN checklist for assessing the methodological quality of studies on measurement properties of health status measurement instruments: an international Delphi study. Qual Life Res.

[21] Santesso N, Barbara AM (2020). Conclusions from surveys may not consider important biases: a systematic survey of surveys. J Clin Epidemiol.

[22] Sequeira T, Monteiro E, Carvalho L, Silveira A (2017). 10-year experience: routine assessment of health-related quality of life in head and neck cancer patients. Glob J Otolaryngol.

[23] Page MJ, McKenzie JE, Bossuyt PM, Boutron I, Hoffmann TC, Mulrow CD, Shamseer L, Tetzlaff JM, Akl EA, Brennan SE, Chou R, Glanville J, Grimshaw JM, Hróbjartsson A, Lalu MM, Li T, Loder EW, Mayo-Wilson E, McDonald S, McGuinness LA, Stewart LA, Thomas J, Tricco AC, Welch VA, Whiting P, Moher D (2021). The PRISMA 2020 statement: an updated guideline for reporting systematic reviews. BMJ.

[24] Page MJ, Moher D, Bossuyt PM, Boutron I, Hoffmann TC, Mulrow CD, Shamseer L, Tetzlaff JM, Akl EA, Brennan SE, Chou R, Glanville J, Grimshaw JM, Hróbjartsson A, Lalu MM, Li T, Loder EW, Mayo-Wilson E, McDonald S, McGuinness LA, Stewart LA, Thomas J, Tricco AC, Welch VA, Whiting P, McKenzie JE (2021). PRISMA 2020 explanation and elaboration: updated guidance and exemplars for reporting systematic reviews. BMJ.

[25] Repositório de Instrumentos de Medição e Avaliação em Saúde. rimas.uc.pt. Accessed 14 Nov 2020.

[26] Center for Health Studies and Research of the University of Coimbra. https://www.uc.pt/org/ceisuc/CEISUC_. Accessed 15 Nov 2020.

[27] World Health Organization Quality of Life. WHOQOL: measuring quality of life. https://www.who.int/tools/whoqol. Accessed 17 Dec 2020.

[28] Multinational Association of Supportive Care in Cancer (MASCC). www.mascc.org. Accessed 10 Feb 2021.

[29] World Health Organization. https://www.who.int. Accessed 15 Sept 2020.

[30] International Society for Quality of Life. 2021. https://www.isoqol.org/what-is-qol/. Accessed 15 Dec 2020.

[31] Silveira A, Monteiro E, Sequeira T (2018). Head and neck cancer: improving patient-reported outcome measures for clinical practice. Curr Treat Options Oncol.

[32] Basch E (2014). The rationale for collecting patient-reported symptoms during routine chemotherapy. Am Soc Clin Oncol Educ Book Am Soc Clin Oncol Annu Meet.

[33] Basch E, Abernethy AP, Mullins CD, Reeve BB, Smith ML, Coons SJ, Sloan J, Wenzel K, Chauhan C, Eppard W, Frank ES, Lipscomb J, Raymond SA, Spencer M, Tunis S (2012). Recommendations for incorporating patient-reported outcomes into clinical comparative effectiveness research in adult oncology. J Clin Oncol.

[34] Doolin JW, Halpin M, Berry JL, Hshieh T, Zerillo JA (2020). Why focus on patient-reported outcome measures in older colorectal cancer patients?. Eur J Surg Oncol.

[35] Takes RP, Halmos GB, Ridge JA (2020). Value and quality of care in head and neck oncology. Curr Oncol Rep.

[36] Graupner C, Kimman ML, Mul S, Slok AHM, Claessens D, Kleijnen J, Dirksen CD, Breukink SO (2021). Patient outcomes, patient experiences and process indicators associated with the routine use of patient-reported outcome measures (PROMs) in cancer care: a systematic review. Support Care Cancer.

[37] Sosnowski R, Kulpa M, Kosowicz M, Presicce F, Porpiglia F, Tubaro A, Nunzio CDE, Demkow T (2017). Basic methods for the assessment of health-related quality of life in uro-oncological patients. Minerva Urol Nefrol.

[38] Deliu N, Cottone F, Collins GS, Anota A, Efficace F (2018). Evaluating methodological quality of Prognostic models Including Patient-reported HeAlth outcomes iN oncologY (EPIPHANY): a systematic review protocol. BMJ Open.

[39] Silva J, Silvera A, Scau A, Monteiro E, Sequeira T (2019). Head and neck cancer early identification of malnutrition high risk patients and quality of life optimization. Int J Otolaryngol Head Neck Surg.

[40] Efficace F, Collins GS, Cottone F, Giesinger JM, Sommer K, Anota A, Schlussel MM, Fazi P, Vignetti M (2021). Patient-Reported Outcomes as Independent Prognostic Factors for Survival in Oncology: Systematic Review and Meta-Analysis. Value Health.

[41] Kouzy R, Abi Jaoude J, Lin D, Nguyen ND, El Alam MB, Ludmir EB, Taniguchi CM (2020). Patient-reported outcome measures in pancreatic cancer receiving radiotherapy. Cancers (Basel).

[42] Van Cutsem E, De Gramont A, Henning G, Rougier P, Bonnetain F, Seufferlein T (2017). Improving outcomes in patients with CRC: the role of patient reported outcomes—an ESDO report. Cancers (Basel).

[43] Ahmed S, Barbera L, Bartlett SJ, Bebb DG, Brundage M, Bryan S, Cheung WY, Coburn N, Crump T, Cuthbertson L, Howell D, Klassen AF, Leduc S, Li M, Mayo NE, McKinnon G, Olson R, Pink J, Robinson JW, Santana MJ, Sawatzky R, Moxam RS, Sinclair S, Servidio-Italiano F, Temple W (2020). A catalyst for transforming health systems and person-centred care: Canadian national position statement on patient-reported outcomes. Curr Oncol.

[44] van den Beuken-van Everdingen MHJ, Hochstenbach LMJ, Joosten EAJ, Tjan-Heijnen VCG, Janssen DJA (2016). Update on prevalence of pain in patients with cancer: systematic review and meta-analysis. J Pain Symptom Manag.

[45] Huang I-C, Lee JL, Ketheeswaran P, Jones CM, Revicki DA, Wu AW (2017). Does personality affect health-related quality of life? A systematic review. PLoS ONE.

[46] Sodergren SC, Husson O, Robinson J, Rohde GE, Tomaszewska IM, Vivat B, Dyar R, Darlington AS, EORTC Quality of Life Group (2017). Systematic review of the health-related quality of life issues facing adolescents and young adults with cancer. Qual Life Res.

[47] Antunes B, Harding R, Higginson IJ (2014). Implementing patient-reported outcome measures in palliative care clinical practice: a systematic review of facilitators and barriers. Palliat Med.

[48] Tzelepis F, Rose SK, Sanson-Fisher RW, Clinton-McHarg T, Carey ML, Paul CL (2014). Are we missing the Institute of Medicine’s mark? A systematic review of patient-reported outcome measures assessing quality of patient-centered cancer care. BMC Cancer.

[49] van Egdom LSE, Oemrawsingh A, Verweij LM, Lingsma HF, Koppert LB, Verhoef C, Klazinga NS, Hazelzet JA (2019). Implementing patient-reported outcome measures in clinical breast cancer care: a systematic review. Value Health.

[50] Girgis A, Durcinoska I, Koh E-S, Ng W, Arnold A, Delaney GP (2018). Development of health pathways to standardize cancer care pathways informed by patient-reported outcomes and clinical practice guidelines. JCO Clin Cancer Inform.

[51] Catt S, Starkings R, Shilling V, Fallowfield L (2017). Patient-reported outcome measures of the impact of cancer on patients’ everyday lives: a systematic review. J Cancer Surviv.

[52] Pawloski PA, Brooks GA, Nielsen ME, Olson-Bullis BA (2019). A systematic review of clinical decision support systems for clinical oncology practice. J Natl Compr Cancer Netw.

[53] Silveira A, Monteiro E, Gonçalves J, Sequeira T. Head and neck cancer patient reported outcomes integration in clinical decision: epidemiological perspectives and health related quality of life assessment. Saúde em Foco: Temas Contemporâneos; 2020.

[54] Kotronoulas G, Kearney N, Maguire R, Harrow A, Di Domenico D, Croy S, MacGillivray S (2014). What is the value of the routine use of patient-reported outcome measures toward improvement of patient outcomes, processes of care, and health service outcomes in cancer care? A systematic review of controlled trials. J Clin Oncol.

[55] Hjollund NHI, Valderas JM, Kyte D, Calvert MJ (2019). Health data processes: a framework for analyzing and discussing efficient use and reuse of health data with a focus on patient-reported outcome measures. J Med Internet Res.

[56] Oosting SF, Haddad RI (2019). Best practice in systemic therapy for head and neck squamous cell carcinoma. Front Oncol.

[57] Bland JS (2020). What is the best way to assess functional health? The history of the development and application of the patient reported outcome measurement information system (PROMIS). Integr Med (Encinitas).

[58] Linendoll N, Saunders T, Burns R, Nyce JD, Wendell KB, Evens AM, Parsons SK (2016). Health-related quality of life in Hodgkin lymphoma: a systematic review. Health Qual Life Outcomes.

[59] Mierzynska J, Piccinin C, Pe M, Martinelli F, Gotay C, Coens C, Mauer M, Eggermont A, Groenvold M, Bjordal K, Reijneveld J, Velikova G, Bottomley A (2019). Prognostic value of patient-reported outcomes from international randomised clinical trials on cancer: a systematic review. Lancet Oncol.

[60] Kerrigan K, Patel SB, Haaland B, Ose D, Weinberg Chalmers A, Haydell T, Meropol NJ, Akerley W (2020). Prognostic significance of patient-reported outcomes in cancer. JCO Oncol Pract.

[61] Agarwal JP, Chakraborty S, Laskar SG, Mummudi N, Patil VM, Prabhash K, Noronha V, Purandare N, Joshi A, Tandon S, Arora J, Badhe R (2017). Prognostic value of a patient-reported functional score versus physician-reported Karnofsky Performance Status Score in brain metastases. Ecancermedicalscience.

[62] Barney BJ, Wang XS, Lu C, Liao Z, Johnson VE, Cleeland CS, Mendoza TR (2013). Prognostic value of patient-reported symptom interference in patients with late-stage lung cancer. Qual Life Res.

[63] Husson O, de Rooij BH, Kieffer J, Oerlemans S, Mols F, Aaronson NK, van der Graaf WTA, van de Poll-Franse LV (2020). The EORTC QLQ-C30 summary score as prognostic factor for survival of patients with cancer in the "Real-World": results from the population-based PROFILES registry. Oncologist.

[64] Dunne RF, Loh KP, Williams GR, Jatoi A, Mustian KM, Mohile SG (2019). Cachexia and sarcopenia in older adults with cancer: a comprehensive review. Cancers (Basel).

[65] Clarijs ME, Thurell J, Kühn F, Uyl-de Groot CA, Hedayati E, Karsten MM, Jager A, Koppert LB (2021). Measuring quality of life using patient-reported outcomes in real-world metastatic breast cancer patients: the need for a standardized approach. Cancers (Basel).

[66] Coomans MB, Peeters MCM, Koekkoek JAF, Schoones JW, Reijneveld J, Taphoorn MJB, Dirven L (2020). Research objectives, statistical analyses and interpretation of health-related quality of life data in glioma research: a systematic review. Cancers (Basel).

[67] Fallowfield L, Jenkins V (2015). Psychosocial/survivorship issues in breast cancer: are we doing better?. J Natl Cancer Inst.

[68] Sequeira T, Ferreira PL, Teixeira J, Peres I, Oliveira J, Silveira A (2015). Patient-reported outcomes in prostate cancer: prospective changes analysis for prognosis prediction. J Cancer Ther.

[69] Lee M-K, Oh J (2021). Patient-reported outcomes of regular aerobic exercise in gastric cancer. Cancers (Basel).

[70] Cheng X, Wei S, Zhang H, Xue S, Wang W, Zhang K (2018). Nurse-led interventions on quality of life for patients with cancer: a meta-analysis. Medicine (Baltimore).

[71] Basch E, Barbera L, Kerrigan CL, Velikova G (2018). Implementation of patient-reported outcomes in routine medical care. Am Soc Clin Oncol Educ book Am Soc Clin Oncol Annu Meet.

[72] Buneviciene I, Mekary RA, Smith TR, Onnela J-P, Bunevicius A (2021). Can mHealth interventions improve quality of life of cancer patients? A systematic review and meta-analysis. Crit Rev Oncol Hematol.

[73] Hauth F, Bizu V, App R, Lautenbacher H, Tenev A, Bitzer M, Malek NP, Zips D, Gani C (2019). Electronic patient-reported outcome measures in radiation oncology: initial experience after workflow implementation. JMIR Mhealth Uhealth.

[74] Tevis SE, James TA, Kuerer HM, Pusic AL, Yao KA, Merlino J, Dietz J (2018). Patient-reported outcomes for breast cancer. Ann Surg Oncol.

[75] van Sluis KE, van der Molen L, van Son RJJH, Hilgers FJM, Bhairosing PA, van den Brekel MWM (2018). Objective and subjective voice outcomes after total laryngectomy: a systematic review. Eur Arch Otorhinolaryngol.

[76] Hamann HA, Shen MJ, Thomas AJ, Craddock Lee SJ, Ostroff JS (2018). Development and preliminary psychometric evaluation of a patient-reported outcome measure for lung cancer stigma: the lung cancer stigma inventory (LCSI). Stigma Health.

[77] Leahy AB, Feudtner C, Basch E (2018). Symptom monitoring in pediatric oncology using patient-reported outcomes: why, how, and where next. Patient.

[78] Biber J, Ose D, Reese J (2018). Patient reported outcomes—experiences with implementation in a University Health Care setting. J Patient Rep Outcomes.

[79] Ream E, Hughes AE, Cox A, Skarparis K, Richardson A, Pedersen VH, Wiseman T, Forbes A, Bryant A (2020). Telephone interventions for symptom management in adults with cancer. Cochrane Database Syst Rev.

[80] Jensen RE, Snyder CF, Abernethy AP, Basch E, Potosky AL, Roberts AC, Loeffler DR, Reeve BB (2014). Review of electronic patient-reported outcomes systems used in cancer clinical care. J Oncol Pract.

[81] Dobrozsi S, Panepinto J (2015). Patient-reported outcomes in clinical practice. Hematol Am Soc Hematol Educ Progr.

[82] Karamanidou C, Natsiavas P, Koumakis L, Marias K, Schera F, Schäfer M, Payne S, Maramis C (2020). Electronic patient-reported outcome-based interventions for palliative cancer care: a systematic and mapping review. JCO Clin Cancer Inform.

[83] Lopez G, Liu W, Milbury K, Spelman A, Wei Q, Bruera E, Cohen L (2017). The effects of oncology massage on symptom self-report for cancer patients and their caregivers. Support Care Cancer.

[84] Li J, Luo X, Cao Q, Lin Y, Xu Y, Li Q (2020). Communication needs of cancer patients and/or caregivers: a critical literature review. J Oncol.

[85] Bradley CJ (2019). Economic burden associated with cancer caregiving. Semin Oncol Nurs.

[86] Fiteni F, Le RI, Ousmen A, Isambert N, Anota A, Bonnetain F (2019). Health-related quality of life as an endpoint in oncology phase I trials: a systematic review. BMC Cancer.

[87] Tetar SU, Bruynzeel AME, Lagerwaard FJ, Slotman BJ, Bohoudi O, Palacios MA (2019). Clinical implementation of magnetic resonance imaging guided adaptive radiotherapy for localized prostate cancer. Phys imaging Radiat Oncol.

[88] Cramer H, Lauche R, Klose P, Lange S, Langhorst J, Dobos GJ (2017). Yoga for improving health-related quality of life, mental health and cancer-related symptoms in women diagnosed with breast cancer. Cochrane database Syst Rev.

[89] Rha SY, Park Y, Song SK, Lee CE, Lee J (2015). Caregiving burden and the quality of life of family caregivers of cancer patients: the relationship and correlates. Eur J Oncol Nurs.

[90] Görlach MG, Schrage T, Bokemeyer C, Kröger N, Müller V, Petersen C, Betz CS, Krüll A, Schulz H, Bleich C (2020). Implementation analysis of patient reported outcomes (PROs) in oncological routine care: an observational study protocol. Health Qual Life Outcomes.

[91] Rodziewicz TL, Houseman B, Hipskind JE. Medical error reduction and prevention. In: StatPearls. StatPearls Publishing; 2021. 29763131

[92] Van Der Wees PJ, Nijhuis-Van Der Sanden MWG, Ayanian JZ, Black N, Westert GP, Schneider EC (2014). Integrating the use of patient-reported outcomes for both clinical practice and performance measurement: views of experts from 3 countries. Milbank Q.

[93] Müller M, Jürgens J, Redaèlli M, Klingberg K, Hautz WE, Stock S (2018). Impact of the communication and patient hand-off tool SBAR on patient safety: a systematic review. BMJ Open.

[94] Selman LE, Brighton LJ, Hawkins A (2017). The effect of communication skills training for generalist palliative care providers on patient-reported outcomes and clinician behaviors: a systematic review and meta-analysis. J Pain Symptom Manag.

[95] Moore PM, Rivera S, Bravo-Soto GA, Olivares C, Lawrie TA (2018). Communication skills training for healthcare professionals working with people who have cancer. Cochrane Database Syst Rev.

[96] Lloyd-Williams M, Payne S, Reeve J, Dona RK (2014). Thoughts of self-harm and depression as prognostic factors in palliative care patients. J Affect Disord.

[97] Warrington L, Absolom K, Velikova G (2015). Integrated care pathways for cancer survivors—a role for patient-reported outcome measures and health informatics. Acta Oncol (Madr).

[98] Shilling V, Matthews L, Jenkins V, Fallowfield L (2016). Patient-reported outcome measures for cancer caregivers: a systematic review. Qual Life Res.

[99] Plevinsky JM, Gutierrez-Colina AM, Carmody JK, Hommel KA, Crosby LE, McGrady ME, Pai ALH, Ramsey RR, Modi AC (2020). Patient-reported outcomes for pediatric adherence and self-management: a systematic review. J Pediatr Psychol.

[100] Litzelman K (2019). Caregiver well-being and the quality of cancer care. Semin Oncol Nurs.

[101] Bainbridge D, Giruparajah M, Zou H, Seow H (2018). The care experiences of patients who die in residential hospice: a qualitative analysis of the last three months of life from the views of bereaved caregivers. Palliat Support Care.

[102] Hui D, Bruera E (2016). Integrating palliative care into the trajectory of cancer care. Nat Rev Clin Oncol.

[103] Dionne-Odom JN, Azuero A, Lyons KD, Hull JG, Tosteson T, Li Z, Li Z, Frost J, Dragnev KH, Akyar I, Hegel MT, Bakitas MA (2015). Benefits of early versus delayed palliative care to informal family caregivers of patients with advanced cancer: outcomes from the ENABLE III randomized controlled trial. J Clin Oncol.

[104] Baudry A-S, Vanlemmens L, Anota A, Cortot A, Piessen G, Christophe V (2019). Profiles of caregivers most at risk of having unmet supportive care needs: Recommendations for healthcare professionals in oncology. Eur J Oncol Nurs.

[105] Wittenberg E, Buller H, Ferrell B, Koczywas M, Borneman T (2017). Understanding family caregiver communication to provide family-centered cancer care. Semin Oncol Nurs.

[106] Carmel S, Singer Y, Yosef-Sela N, Bachner YG (2020). Open communication between caregivers’ and terminally ill cancer patients about illness and death: the role of gender—a correlational study. Eur J Oncol Nurs.

[107] Roydhouse JK, Wilson IB (2017). Systematic review of caregiver responses for patient health-related quality of life in adult cancer care. Qual Life Res.

[108] Wittenberg E, Xu J, Goldsmith J, Mendoza Y (2019). Caregiver communication about cancer: development of a mhealth resource to support family caregiver communication burden. Psychooncology.

[109] Washington KT, Craig KW, Parker Oliver D, Ruggeri JS, Brunk SR, Goldstein AK, Demiris G (2019). Family caregivers' perspectives on communication with cancer care providers. J Psychosoc Oncol.

[110] Jadalla A, Ginex P, Coleman M, Vrabel M, Bevans M (2020). Family caregiver strain and burden: a systematic review of evidence-based interventions when caring for patients with cancer. Clin J Oncol Nurs.

[111] Goldsmith JV, Wittenberg E, Terui S, Kim H, Umi S (2019). Providing support for caregiver communication burden: assessing the plain language planner resource as a nursing intervention. Semin Oncol Nurs.

[112] Rochfort A, Beirne S, Doran G, Patton P, Gensichen J, Kunnamo I, Smith S, Eriksson T, Collins C (2018). Does patient self-management education of primary care professionals improve patient outcomes: a systematic review. BMC Fam Pract.

[113] Kwan YH, Weng SD, Loh DHF, Phang JK, Oo LJY, Blalock DV, Chew EH, Yap KZ, Tan CYK, Yoon S, Fong W, Østbye T, Low LL, Bosworth HB, Thumboo J (2020). Measurement properties of existing patient-reported outcome measures on medication adherence: systematic review. J Med Internet Res.

[114] Kataria T, Gupta D, Goyal S, Bisht SS, Basu T, Abhishek A, Narang K, Banerjee S, Nasreen S, Sambasivam S, Dhyani A (2016). Clinical outcomes of adaptive radiotherapy in head and neck cancers. Br J Radiol.

[115] Giesinger J, Kemmler G, Meraner V, Gamper EM, Oberguggenberger A, Sperner-Unterweger B, Holzner B (2009). Towards the implementation of quality of life monitoring in daily clinical routine: methodological issues and clinical implication. Breast Care (Basel).

[116] Silveira A, Gonçalves J, Sequeira T, Ribeiro C, Lopes C, Monteiro E, Pimentel FL (2012). Oncologia de Cabeça e Pescoço: enquadramento epidemiológico e clínico na avaliação da Qualidade de Vida Relacionada com a Saúde [Head and neck cancer: health related quality of life assessment considering clinical and epidemiological perspectives]. Rev Bras Epidemiol.

[117] Kovic B, Jin X, Kennedy SA, Hylands M, Pedziwiatr M, Kuriyama A, Gomaa H, Lee Y, Katsura M, Tada M, Hong BY, Cho SM, Hong PJ, Yu AM, Sivji Y, Toma A, Xie L, Tsoi L, Waligora M, Prasad M, Bhatnagar N, Thabane L, Brundage M, Guyatt G, Xie F (2018). Evaluating progression-free survival as a surrogate outcome for health-related quality of life in oncology: a systematic review and quantitative analysis. JAMA Intern Med.

[118] Jensen RE, Rothrock NE, DeWitt EM, Spiegel B, Tucker CA, Crane HM, Forrest CB, Patrick DL, Fredericksen R, Shulman LM, Cella D, Crane PK (2015). The role of technical advances in the adoption and integration of patient-reported outcomes in clinical care. Med Care.

[119] Philipson RG, Wu AD, Curtis WC, Jablonsky DJ, Hegde JV, McCloskey SA, Kaprealian TB, Steinberg ML, Kishan AU, Raldow AC (2021). A practical guide for navigating the design, build, and clinical integration of electronic patient-reported outcomes in the Radiation Oncology Department. Pract Radiat Oncol.

[120] Concannon TW, Fuster M, Saunders T, Patel K, Wong JB, Leslie LK, Lau J (2014). A systematic review of stakeholder engagement in comparative effectiveness and patient-centered outcomes research. J Gen Intern Med.

[121] Gordon RJ, Mikles SP, Kneale L, Evans HL, Munson SA, Backonja U, Lober WB (2020). How patient-generated health data and patient-reported outcomes affect patient-clinician relationships: A systematic review. Health Inform J.

[122] Hsiao C-J, Dymek C, Kim B, Russell B (2019). Advancing the use of patient-reported outcomes in practice: understanding challenges, opportunities, and the potential of health information technology. Qual Life Res.

[123] Alsaleh K (2013). Routine administration of standardized questionnaires that assess aspects of patients’ quality of life in medical oncology clinics: a systematic review. J Egypt Natl Cancer Inst.

[124] Zheng M, Jin H, Shi N, Duan C, Wang D, Yu X, Li X (2018). The relationship between health literacy and quality of life: a systematic review and meta-analysis. Health Qual Life Outcomes.

[125] Proctor EK, Powell BJ, McMillen JC (2013). Implementation strategies: recommendations for specifying and reporting. Implement Sci.

[126] Gray-Burrows KA, Willis TA, Foy R, Rathfelder M, Bland P, Chin A, Hodgson S, Ibegbuna G, Prestwich G, Samuel K, Wood L, Yaqoob F, McEachan R (2018). Role of patient and public involvement in implementation research: a consensus study. BMJ Qual Saf.

