# Supplementary material for: Patient reported outcomes in oncology: changing perspectives—a systematic review
Source: Health Qual Life Outcomes. 2022 May 21;20:82. doi: 10.1186/s12955-022-01987-x (PMC9124403; doi:10.1186/s12955-022-01987-x)
Supplement: Supplementary file 1 — Additional file 1. Summary of studies’ characteristics. [file 12955_2022_1987_MOESM1_ESM.docx]

**Table S1.** Summary of studies’ characteristics

| **Study** | **Ref** | **Type of Manuscript** | **Oncology Patient** | | | | | **Caregivers** | | | | **Health Systems, Communities & Society** | | | | **Critical Success Factors** | | | | | **Implementation Methodology** | | | | | **Routine Clinical Practice** | | | |  |  |  |
| --- | --- | --- | --- | --- | --- | --- | --- | --- | --- | --- | --- | --- | --- | --- | --- | --- | --- | --- | --- | --- | --- | --- | --- | --- | --- | --- | --- | --- | --- | --- | --- | --- |
|  |  |  | **A1** | **A2** | **A3** | **A4** | **A5** | **B1** | **B2** | **B3** | **B4** | **C1** | **C2** | **C3** | **C4** | **D1** | **D2** | **D3** | **D4** | **D5** | **E1** | **E1** | **E3** | **E4** | **E5** | **F1** | **F2** | **F3** | **F4** |  |  |  |
| Agarwal *et al,* 2017 | 61 | RA |  | × |  | × | × |  |  |  |  |  |  |  |  |  |  |  |  |  |  |  |  |  |  |  |  |  |  |  |  |  |
| Ahmed *et al,* 2020 | 43 | RP | × | × | × | × | × | × | × |  |  | × |  | × | × | × | × | × | × | × |  |  |  |  |  | × | × |  |  |  |  |  |
| Alsaleh,2013 | 123 | SR |  |  |  |  |  |  |  |  |  |  |  |  |  | × |  |  |  | × |  | × |  |  |  | × |  | × |  |  |  |  |
| Antune*s* et al, 2014 | 47 | SR | × | × | × | × |  |  |  |  |  |  | × |  | × |  |  |  |  |  |  |  |  |  |  | × |  | × |  |  |  |  |
| Austin *et al,* 2020 | 10 | RA |  |  | × | × | × |  |  |  |  |  |  |  | × | × |  | × | × | × |  | × | × | × |  | × |  | × |  |  |  |  |
| Barney *et al, 2013* | 62 | RP |  | × |  | × | × |  |  |  |  |  |  |  |  |  |  |  |  |  |  |  |  |  |  |  |  |  |  |  |  |  |
| Basch *et al,* 2018 | 71 | RP |  | × | × |  | × |  |  |  |  |  |  |  |  | × | × | × | × | × | × | × | × |  |  | × | × |  |  |  |  |  |
| Basch*,*2014 | 32 | RP | × |  | × |  | × |  |  |  |  |  |  |  | × |  |  | × |  | × | × |  |  |  |  | × | × | × |  |  |  |  |
| Baudry *et al,* 2019 | 104 | RA |  |  |  |  |  |  |  | × | × | × |  |  |  |  |  |  |  |  |  |  |  |  |  | × | × |  |  |  |  |  |
| Biber *et al,* 2018 | 78 | RA |  |  | × | × | × |  |  |  |  | × |  |  | × | × | × | × | × | × | × | × | × |  |  | × | × |  |  |  |  |  |
| Bland*,*2020 | 57 | RP | × | × |  |  |  |  |  |  |  | × |  |  | × |  |  |  |  |  |  |  |  |  |  |  |  |  |  |  |  |  |
| Bradley,2019 | 85 | RP |  |  |  |  |  |  |  |  | × | × | × |  |  |  |  |  |  |  |  |  |  |  |  |  |  |  |  |  |  |  |
| Buneviciene *et al,* 2021 | 72 | SR |  | × |  |  | × |  |  |  |  |  |  |  | × |  |  |  |  |  |  |  |  |  |  | × |  |  | × |  |  |  |
| Carmel *et al,* 2020 | 106 | RA |  |  |  |  |  |  |  | × |  | × |  |  |  |  |  |  |  |  |  |  |  |  |  | × |  |  | × |  |  |  |
| Catt *et al,* 2017 | 51 | SR | × |  |  |  | × |  |  |  |  |  |  |  | × |  |  |  |  |  |  |  |  |  |  |  |  |  |  |  |  |  |
| Cheng *et al,* 2018 | 70 | SR |  | × |  | × |  |  |  |  |  |  |  |  | × |  |  |  |  |  |  |  |  |  |  |  |  |  |  |  |  |  |
| Clarijs *et al,* 2021 | 65 | RP |  | × | × | × | × |  |  |  |  | × |  |  |  |  |  |  |  |  |  |  |  |  |  | × |  |  | × |  |  |  |
| Coomans *et al,* 2020 | 66 | SR |  | × | × | × | × |  |  |  |  | × |  |  |  |  |  |  |  |  |  |  |  |  |  |  |  |  |  |  |  |  |
| Cramer *et al,* 2017 | 88 | RP |  |  |  |  | × |  |  |  |  |  |  |  |  |  |  |  |  |  |  |  |  |  |  |  |  |  |  |  |  |  |
| Deliu *et al,* 2018 | 38 | SR | × | × | × |  |  |  |  |  |  | × |  |  | × |  |  |  |  |  |  |  |  |  |  | × |  |  |  |  |  |  |
| Dionne-Odom *et al,* 2015 | 103 | RA |  |  |  |  |  |  |  | × | × | × |  |  | × |  |  |  |  |  |  |  |  |  |  |  |  |  |  |  |  |  |
| Dobrozsi&Panepinto,2015 | 81 | RP |  |  | × | × | × |  |  |  |  |  |  |  |  | × | × | × | × | × | × | × | × | × |  | × |  |  |  |  |  |  |
| Doolin *et al,* 2020 | 34 | RP | × | × | × | × | × |  |  |  |  | × |  |  | × |  |  |  |  |  |  |  |  |  |  |  |  |  |  |  |  |  |
| Dunne *et al,* 2019 | 64 | RP |  | × |  | × | × |  |  |  |  |  |  |  | × |  |  |  |  |  |  |  |  |  |  |  |  |  |  |  |  |  |
| Efficace *et al,* 2021 | 40 | SR | × | × | × | × | × |  |  |  |  |  |  | × | × |  |  |  |  |  |  |  |  |  |  |  |  |  |  |  |  |  |
| Fallowfield&Jenkins,2015 | 67 | RP |  | × | × |  |  |  |  |  |  |  |  |  | × |  |  |  |  |  |  |  |  | × |  |  |  |  | × |  |  |  |
| Fiteni *et al,* 2019 | 86 | SR |  |  |  | × |  |  |  |  |  | × |  | × |  |  |  |  |  |  |  |  |  |  |  |  |  |  | × |  |  |  |
| Girgis *et al,* 2018 | 50 | RP | × | × | × | × | × |  |  |  |  |  |  |  |  |  |  | × | × | × | × | × | × |  |  | × | × |  | × |  |  |  |
| Görlach *et al,* 2020 | 90 | RA |  |  |  |  | × |  |  |  |  |  |  |  |  | × |  | × | × | × | × | × | × | × | × | × |  | × | × |  |  |  |
| Graupner *et al,* 2021 | 36 | SR | × |  | × | × | × |  |  |  | × |  |  |  | × |  |  | × |  |  |  |  |  |  |  |  |  |  | × |  |  |  |
| Hamann *et al,* 2018 | 76 | RA |  |  | × |  | × |  |  |  |  |  |  |  |  |  |  |  |  |  |  |  |  |  |  |  |  |  | × |  |  |  |
| Hauth *et al,* 2019 | 73 | RA |  | × | × | × | × |  |  |  |  | × |  |  | × | × |  | × |  |  |  |  |  |  |  | × | × |  | × |  |  |  |
| Hjollund *et al,* 2019 | 55 | RP | × |  | × | × | × |  |  |  |  | × |  |  | × |  |  | × |  | × | × | × | × |  |  | × | × |  | × |  |  |  |
| Huang *et al,* 2017 | 45 | SR |  |  |  |  | × |  |  |  |  |  |  |  |  |  |  |  |  |  |  |  |  |  |  | × | × |  | × |  |  |  |
| Hui&Bruera,2016 | 100 | RP |  |  |  |  |  |  | × | × | × | × |  |  | × |  |  |  |  |  |  |  |  |  |  | × |  |  | × |  |  |  |
| Husson *et al,* 2020 | 63 | RA |  | × |  |  |  |  |  |  |  | × |  |  |  |  |  |  |  |  |  |  |  |  |  |  | × |  |  |  |  |  |
| Jadalla *et al,* 2020 | 110 | SR |  |  |  |  |  |  |  | × | × | × |  |  | × |  |  |  |  |  |  |  |  |  |  |  |  |  |  |  |  |  |
| Jensen *et al,* 2014 | 80 | RP |  |  | × | × |  |  |  |  |  |  |  |  | × | × | × | × |  | × |  | × | × | × |  | × |  |  |  |  |  |  |
| Jensen *et al,* 2015 | 118 | RP |  |  |  |  |  |  |  |  |  |  |  |  |  | × | × | × | × | × | × | × | × | × |  | × | × | × |  |  |  |  |
| Karamanidou *et al,* 2020 | 82 | SR |  |  | × | × | × |  |  |  |  | × |  |  | × | × |  |  |  |  |  |  |  |  |  | × |  |  | × |  |  |  |
| Kerrigan *et al,* 2020 | 60 | RP |  | × |  | × | × |  |  |  |  | × |  |  | × |  |  |  |  |  |  |  |  |  |  | × | × |  | × |  |  |  |
| Kotronoulas *et al,* 2014 | 54 | SR | × |  | × | × | × |  |  |  |  | × |  |  | × |  |  |  |  |  |  |  |  |  |  | × |  | × | × |  |  |  |
| Kouzy *et al,* 2020 | 41 | RP | × | × | × | × | × |  |  |  |  |  |  |  | × |  |  |  |  |  |  |  |  |  |  |  |  |  | × |  |  |  |
| Kovic *et al,* 2018 | 117 | SR |  |  |  |  |  |  |  |  |  |  |  | × |  |  |  |  |  |  |  | × |  |  |  | × |  |  | × |  |  |  |
| Leahy *et al,* 2018 | 77 | RP |  |  | × | × | × |  |  |  |  | × |  |  |  | × |  |  | × | × |  | × | × | × | × | × |  |  | × |  |  |  |
| Le*e et al,* 2021 | 69 | RA |  | × | × |  | × |  |  |  |  | × |  |  | × |  |  |  |  |  |  |  |  |  |  |  |  |  | × |  |  |  |
| Li *et al,* 2020 | 84 | RP |  |  | × | × | × |  |  | × |  | × |  |  |  |  |  |  |  |  |  |  |  |  |  |  |  |  | × |  |  |  |
| Linendoll *et al,* 2016 | 58 | SR |  | × |  |  | × |  |  |  |  |  |  |  | × |  |  |  |  |  |  |  |  |  |  | × |  |  | × |  |  |  |
| Litzelman,2019 | 100 | RP |  |  |  |  |  | × | × | × | × | × |  |  | × |  |  |  |  |  |  |  |  |  |  | × |  |  | × |  |  |  |
| Lopez *et al,* 2017 | 83 | RA |  |  |  | × |  |  |  |  | × |  |  |  | × |  |  |  |  |  |  |  |  |  |  |  |  |  | × |  |  |  |
| Mierzynska *et al,* 2019 | 59 | SR |  | × |  |  | × |  |  |  |  | × |  |  |  |  |  |  |  |  |  |  |  |  |  | × | × |  | × |  |  |  |
| Oosting&Haddad,2019 | 56 | RP | × |  |  | × | × |  |  |  |  |  |  |  | × |  |  |  |  |  |  | × |  |  |  | × |  |  | × |  |  |  |
| Pawloski *et al,* 2019 | 52 | SR | × |  |  | × |  |  |  |  |  | × | × |  | × |  |  |  |  |  |  |  |  |  |  |  |  |  | × |  |  |  |
| Ream *et al,* 2020 | 79 | RP |  |  | × | × | × |  |  |  |  | × |  |  | × |  |  |  |  |  |  |  |  |  |  |  |  |  | × |  |  |  |
| Rha *et al,* 2015 | 89 | RA |  |  |  | × | × | × |  | × | × | × |  |  | × |  |  |  |  |  |  |  |  |  |  | × |  |  |  |  |  |  |
| Ringash *et al,* 2018 | 12 | RP |  |  |  |  | × |  |  | × | × | × |  |  | × |  |  |  |  |  |  |  |  |  |  | × |  |  |  |  |  |  |
| Roydhouse&Wilson,2017 | 107 | SR |  |  |  |  |  | × |  |  | × |  |  |  |  |  |  |  |  |  |  |  |  |  |  |  |  |  |  |  |  |  |
| Sequeira *et al,* 2015 | 68 | RA |  | × | × | × | × |  |  |  |  | × |  |  | × |  |  |  |  |  |  |  |  |  |  |  |  |  |  |  |  |  |
| Sequeira *et al,* 2017 | 22 | RP | × | × | × | × | × |  |  |  |  | × |  |  | × | × | × | × |  |  | × | × |  |  |  | × | × |  | × |  |  |  |
| Sequeira *et al,* 2020 | 2 | RP |  | × |  |  | × |  |  |  |  |  |  |  | × |  |  |  |  |  |  |  |  |  |  |  |  |  | × |  |  |  |
| Shilling *et al,* 2016 | 98 | SR |  |  |  |  |  |  | × |  | × |  |  |  |  |  |  |  |  |  |  |  |  |  |  |  |  |  | × |  |  |  |
| Shrestha *et al, 2019* | 3 | SR |  | × |  |  |  |  |  |  |  | × |  |  |  |  |  |  |  |  |  |  |  |  |  | × |  |  | × |  |  |  |
| Silva *et al, 2019* | 39 | RA | × | × |  | × | × |  |  |  |  |  |  |  |  |  |  |  |  |  |  |  |  |  |  |  |  |  |  |  |  |  |
| Silveira *et al,* 2018a | 16 | RA |  |  |  |  |  | × | × | × | × | × |  |  | × |  |  |  |  |  |  |  |  |  |  | × |  |  |  |  |  |  |
| Silveira *et al,* 2018b | 31 | RP | × | × | × | × | × |  |  |  |  | × | × |  | × | × | × | × | × | × | × | × | × |  |  | × | × |  | × |  |  |  |
| Silveira *et al,* 2020a | 4 | RP | × | × | × | × | × |  |  |  |  | × |  |  | × |  |  |  |  |  |  |  |  |  |  | × |  |  | × |  |  |  |
| Silveira *et al,* 2020b | 53 | RP | × | × | × | × | × |  |  |  |  | × |  |  | × |  |  |  |  |  |  |  |  |  |  | × |  |  | × |  |  |  |
| Sodergren *et al,* 2017 | 46 | SR | × |  | × |  | × |  |  |  |  |  |  |  |  |  |  |  |  |  |  |  |  |  |  |  |  |  | × |  |  |  |
| Sosnowski *et al,* 2017 | 37 | SR | × | × |  |  | × |  |  |  |  |  |  |  | × | × |  |  |  |  |  |  |  |  |  | × |  | × | × |  |  |  |
| Takes *et al,* 2020 | 35 | RP |  |  |  |  |  |  |  |  |  |  |  |  |  |  |  |  |  |  |  |  |  |  |  |  |  |  |  |  |  |  |
| Tetar *et al,* 2019 | 87 | RA |  |  |  | × |  |  |  |  |  |  |  |  |  |  |  |  |  |  |  | × |  |  |  | × |  |  | × |  |  |  |
| Tevis *et al,* 2018 | 74 | RP |  | × | × | × | × |  |  |  |  |  |  |  | × | × | × | × | × | × | × | × | × | × |  | × | × |  |  |  |  |  |
| Tran *et al,* 2018 | 5 | RP | × |  | × | × | × |  |  |  |  | × |  |  | × |  | × | × |  |  |  | × |  |  |  | × |  |  | × |  |  |  |
| Tzelepis *et al,* 2014 | 48 | SR | × |  | × | × |  |  |  |  |  | × |  |  | × |  |  |  |  |  |  |  |  |  |  |  | × |  | × |  |  |  |
| Ullrich *et al,* 2017 | 17 | RA |  |  |  |  |  |  |  | × | × |  |  |  |  |  |  |  |  |  |  |  |  |  |  |  |  |  | × |  |  |  |
| VanCutsem *et al, 2017* | 42 | RP | × | × | × | × | × |  |  |  |  |  |  |  | × |  |  |  |  |  |  |  |  |  |  | × | × |  | × |  |  |  |
| vandenBeuken *et al,* 2016 | 44 | SR | × |  |  |  |  |  |  |  |  | × |  |  | × |  |  |  |  |  |  |  |  |  |  |  |  |  | × |  |  |  |
| vanEgdom *et al,* 2019 | 49 | SR | × |  | × |  | × |  |  |  |  |  |  |  | × | × |  |  |  |  |  | × |  |  |  | × |  |  | × |  |  |  |
| vanSluis *et al,* 2018 | 75 | SR | × |  | × |  | × |  |  |  |  |  |  |  |  |  |  |  |  |  |  |  |  |  |  |  |  |  | × |  |  |  |
| Warrington *et al,* 2015 | 97 | RP |  |  |  |  |  |  |  |  |  |  |  |  |  | × | × | × | × | × | × | × | × |  |  | × | × |  |  |  |  |  |
| Washington *et al,* 2019 | 109 | RA |  |  |  |  |  |  |  | × |  | × |  |  | × |  |  |  |  |  |  |  |  |  |  |  |  |  | × |  |  |  |
| Wittenberg *et al,* 2017 | 105 | RA |  |  |  |  |  |  |  | × |  | × |  |  |  |  |  |  |  |  |  |  |  |  |  |  |  |  | × |  |  |  |
| Wittenberg *et al,* 2019 | 108 | RA |  |  |  |  |  | × | × |  | × |  |  |  | × |  |  |  |  |  |  |  |  |  |  |  |  |  | × |  |  |  |

Legend: Type of Manuscript - Systematic Review (SR); Research article (RA); Retrospective, prospective (RP)

Oncology Patient - Stepped care models (A1); Prognostic value (A2); Communication (A3); Patient safety (A4); Risk identification (A5)

Caregivers - Communication improvements (B1); Supportive care guidance (B2); Caregiver support (B3); Caregiver burden (B4)

Health systems, communities & society - Futures perspectives (C1); Economical decisions (C2); Drug approval (C3); Patient centered care (C4)

Critical success factors - Staff involvement (D1); Institution approval (D2); Clear workflows (D3); Team continuous formation (D4); Data analysis (D5)

Implementation methodology - Research intervention (E1); Clinical Interventions (E2); Medical Interventions (E3); Physiological interventions (E4); Social Interventions (E5)

Routine clinical practice - Use for clinical purposes (F1); Use for research purposes (F2); Use for economical purposes (F3); Quality of care (F4)
